# Transcriptomic Analysis Reveals Developmental Toxicity Induced by Environmentally Relevant Concentrations of Fenvalerate and Sulfamethoxazole in Embryo and Juvenile Marine Medaka (*Oryzias melastigma*, McClelland, 1839)

**DOI:** 10.3390/ani15182765

**Published:** 2025-09-22

**Authors:** Shuyuan Chen, Meina Duan, Qian Liu, Yuna Huang, Dong Sun

**Affiliations:** 1 College of Chemistry and Environment, Guangdong Ocean University, Zhanjiang 524088, China; 13068984243@163.com (S.C.); duanmeinahuanjing@163.com (M.D.); 2023129252123@stu.gdou.edu.cn (Q.L.); igeedz@163.com (Y.H.); 2 Zhanjiang Key Laboratory of Pollutant Control and Ecological Restoration for Coastal Marine Aquaculture, Guangdong Ocean University, Zhanjiang 524088, China; 3 Research Center for Coastal Environmental Protection and Ecological Resilience, Guangdong Ocean University, Zhanjiang 524088, China

**Keywords:** fenvalerate, sulfamethoxazole, marine medaka, transcriptomic analysis, synergistic toxicity

## Abstract

The abuse of pesticides and antibiotics has increased the potential risks for coastal marine organisms. Thus, the evaluation of the toxic effects on and mechanisms of both marine organisms is very important. This study investigated the developmental toxicity and mechanisms of FEN and SMX in embryo and juvenile marine medaka. The results showed that when embryos were exposed to FEN (0.3, 30 μg·L^−1^) or SMX (1, 10 μg·L^−1^) for 12 days, there were no significant effects on hatching, malformation, or mortality rates. However, the combined exposure to F-S (F-0.3 + S-10) significantly reduced the hatching rate and increased the malformation rate and mortality rate. Histological analysis revealed that there were no pathological changes in the tissues of juvenile marine medaka, indicating no sensitivity at low doses. A transcriptome analysis of tissues exposed to F-S identified 1135 differentially expressed genes (DEGs), which was significantly more than that for single exposure groups (FEN: 499 DEGs; SMX: 138 DEGs). KEGG enrichment analysis highlighted that growth-related pathways were disturbed, such as steroid biosynthesis, the cell cycle, fatty acid metabolism, etc. Our findings suggest that the combined exposure to FEN and SMX induces developmental toxicity by regulating the dysregulation of metabolic and proliferative pathways, highlighting the ecological risks posed to marine organisms in polluted estuaries.

## Simple Summary

The long-term abuse of pesticides and antibiotics has become a hot topic, and their concentrations in coastal environments may have already caused harm to aquatic organisms. To address this issue, we selected two common pesticides (fenvalerate) and antibiotics (sulfamethoxazole) to conduct exposure tests on the embryonic and juvenile stages of marine medaka. The results showed that when each chemical was tested alone, it did not cause serious problems such as hatching failure, deformity, or death. However, when the two substances were used in combination, the situation became much worse: the number of hatched fish eggs decreased, the number of deformed fish increased, and more fish died. We also examined the internal tissues of the fish and found no damage, but when we looked at their genes, we discovered significant changes—especially in genes related to growth and metabolism. This indicates that the combined use of these chemicals can cause harm to marine organisms, which is crucial for protecting our marine environment.

## 1. Introduction

Human activities are increasingly impacting the marine environment, raising growing concern about the threats from emerging environmental pollutants to marine organisms and ecosystems. Fenvalerate (FEN) and sulfamethoxazole (SMX), two prominent environmental pollutants, are continuously introduced into the marine environment through various sources such as surface runoff, aquaculture tail water discharge, industrial and agricultural sewage discharge, and underground rivers. Studies have shown that tens of thousands of tons of antibiotics and pesticides enter offshore areas annually, and they are detected in offshore organisms and coastal residents [1].

FEN, a widely used low-toxic pesticide in agriculture, is a known carcinogen with potential ecological risks due to its specific toxic effects on fish in aquatic environments [2]. FEN belongs to second-generation pyrethroid insecticides, which disrupt sodium channel function in pest neurons, causing irreversible excitation and paralysis [3]. FEN has become an important agricultural insecticide [4]. It is worth noting that FEN can damage aquatic organisms in specific ways. For instance, 0.0086 mg·L^−1^ of FEN significantly reduces the digestive enzyme activity of zebrafish, induces neurotoxic effects, interferes with the synthesis of fish sex hormones, and causes abnormal gonadal development [5]. A possible mechanism for this is that FEN, as a sodium channel blocker, inhibits acetylcholinesterase (AChE) activity in zebrafish and disrupts neurotransmitter signal transmission [6]. Furthermore, studies have found that FEN also has the ability to cause oxidative damage and induce and alter the physiological and biochemical phenomena of organisms [7,8]. These toxic effects may have very adverse impacts on fish.

Sulfamethoxazole, one of the sulfonamide antibiotics, is a commonly detected environmental pollutant in marine environments, due to its broad-spectrum antibacterial properties and cost-effectiveness [9]. SMX exerts a significant bactericidal effect by inhibiting the enzymatic pathways involved in bacterial folic acid synthesis [10]. Studies have shown that SMX has high bioaccumulation potential [11]. Furthermore, SMX induces immune and inflammatory responses in zebrafish, leading to morphological damage in zebrafish larvae [12], and impairs intestinal barrier function and reduces the expression of intestinal tight junction proteins at 0.3 μg·L^−1^ [13]. Notably, there is growing evidence that SMX at low concentrations (0.26 μg·L^−1^) can cause damage to the immune system of fish and promote the expression of pro-inflammatory cytokines [14], and this phenomenon is more concentrated in the embryo-to-larval stage of fish [15].

Coastal areas and estuaries serve as convergence zones for various pollutants, where they often accumulate in mixed forms. Therefore, there is a critical need to investigate the combined toxic effects of FEN and SMX at environmentally relevant concentrations on marine organisms. FEN and SMX are relatively stable in the environment and difficult to degrade due to their specific physical and chemical properties [16]. Numerous studies have shown that these compounds can bioaccumulate and transfer through food chains in organisms, increasing cancer risk and damaging the antioxidant, nervous, immune, and endocrine systems of organisms [17,18,19,20,21]. Therefore, further research on the combined toxicity of FEN and SMX to marine organisms is needed to assess the risks posed by their combined exposure to both marine life and human health.

Juvenile fish are in a critical stage of organogenesis and functional development, and they are extremely sensitive to external disturbances [22]. This sensitivity makes them ideal for evaluating the toxicological effects of environmentally relevant concentrations. Meanwhile, the combined toxic effects and mechanisms of FEN and SMX at environmental concentrations on fish remain unclear. In this study, the toxic effects of FEN and SMX on embryos and juvenile marine medaka (*Oryzias melastigma*) were evaluated through embryological observation, a histological examination of juvenile fish tissues, transcriptomic analysis, and molecular biology methods. This study revealed the toxicological mechanisms underlying the combined effects of FEN and SMX on growth, development, and endocrine disruption in marine medaka. These results provide foundational data on the impacts of combined FEN and SMX exposure on fish and offer a theoretical basis for assessing and restoring offshore marine ecosystems.

## 2. Materials and Methods

### 2.1. Chemicals and Exposure Concentration Selection

Fenvalerate (CAS: 51630-58-1, purity ≥ 97%) and sulfamethoxazole (CAS:723-46-6, purity ≥ 98%) were purchased from Shanghai Maclin Biochemical Technology Co., Ltd. (Shanghai, China). 

FEN was dissolved with DMSO (Shanghai Maclin Biochemical Technology Co., Ltd., Shanghai, China) to prepare a stock solution with a concentration of 0.6 g·L^−1^, which was stored at room temperature in the dark. SMX was dissolved with DMSO to prepare a stock solution with a concentration of 1 g·L^−1^, which was stored under the same conditions.

The exposure concentrations were the same as those monitored in the actual environment, with FEN at 0.3 and 30 μg·L^−1^ and SMX at 1 and 10 μg·L^−1^, respectively [23,24,25].

### 2.2. Embryo Collection and Juvenile Fish Culture of Marine Medaka

The marine medaka used in this experiment were cultured by the College of Fisheries, Guangdong Ocean University. Mature fertilized eggs from adult marine medaka were collected and cultured. Each pair of adult fish produced 10–50 eggs, all from the same batch. Fertilized eggs were incubated at 26 °C, 30 psu salinity, and a 12:12 light–dark cycle. After approximately 10 days, healthy juveniles were collected. From 1 to 7 days post-hatching, juveniles were fed a yolk suspension. From day 8 to 30 days, they were fed newly hatched *Artemia nauplii* twice daily. After one month, juveniles were transferred to glass tanks for experimentation.

### 2.3. Experimental Design

Embryo Exposure Period: Fertilized embryos were collected, and only those collected between 8:00 and 10:00 am were used to ensure consistent fertilization stages. The experimental groups were set as follows: FEN (0.3 and 30 μg·L^−1^), SMX (1 and 10 μg·L^−1^), and FEN + SMX (F-0.3 + S-1 and F-0.3 + S-10). A blank control group (CK) was included for each pollutant. Thirty embryos were placed in each Petri dish (9 cm), with three replicates per treatment group. Each Petri dish contained 20 mL of exposure solution, which was replaced once daily. Embryonic development was observed, and the numbers of malformed and dead embryos were recorded. After 12 days of exposure, the hatching rate, malformation rate, and mortality were calculated [26].

Juvenile Fish Exposure Period: Groups for FEN (0.3 μg·L^−1^), SMX (1 μg·L^−1^), and FEN + SMX (F-0.3 + S-1, abbreviation as F-S) were established, with a blank control group (CK) for each pollutant. Each fish tank was filled with 10 L of pre-aerated artificial seawater, containing 30 juvenile fish (three replicates per treatment). The exposure period was 15 days, with the temperature controlled at 25–26 °C. And artemia was fed at 12:00 and 18:00 daily. Half of the exposure solution was replaced daily to maintain pollutant concentrations. Feeding was stopped 24 h before the end of the exposure. After 15 days, the body length of juvenile marine medaka was measured using a vernier caliper, and then the fish were frozen in liquid nitrogen and stored at −80 °C for subsequent transcriptome and qPCR experiments.

Hatching rate = (Number of hatched embryos/Total fertilized embryos) × 100%(1)

Malformation rate = (Number of malformed embryos/Total fertilized embryos) × 100%(2)

Mortality rate = (Number of dead embryos/Total fertilized embryos) × 100%(3)

### 2.4. Hematoxylin and Eosin Staining

Histology and stereology were performed as previously described [26]. Briefly, juvenile fish were fixed in 4% paraformaldehyde for 24 h, embedded in paraffin, and sectioned into 5 μm thick slices. Sections were stained with hematoxylin and eosin (H&E), then analyzed and photographed using digital pathological section scanner (LG-S80, Servicebio Inc., Wuhan, China).

### 2.5. Transcriptomic Analysis

Total RNA was extracted using Trizol reagent (Invitrogen Inc., Carlsbad, CA, USA). The quality and integrity of the extracted RNA were evaluated by agarose gel electrophoresis (DYY-6C, Beijing, China) and Agilent 2100 Bioanalyzer (Agilent Inc., Santa Clara, CA, USA). Sequencing was performed on the Illumina NovaSeq 6000 platform (illumina Inc., San Diego, CA, USA), and raw reads were filtered to obtain clean reads. Reference genome alignment analysis was carried out using HISAT2 software (2.2.0). Based on the alignment results of HISAT2, we reconstructed transcripts with Stringtie and calculated the expression levels of all genes in each sample using RSEM (1.2.19). The expression levels of genes were calculated using FPKM. Based on the expression results of each sample, we understood the repeatability among samples and assisted in excluding outlier samples through PCA and the calculation of Pearson correlation coefficients between samples. Then, differential analysis was performed between groups. The obtained clean reads were input into DESeq2 software (1.48.2) for differential analysis. Based on the results of differential analysis, genes with Q < 0.05 and |log2FC| > 1 were screened as significantly differentially expressed genes. Genes with *p* < 0.05 were identified as significantly differentially expressed, and those with *p* < 0.01 were extremely significantly differentially expressed. RT-qPCR verification was performed for significantly differentially expressed genes.

### 2.6. Quantitative Real-Time PCR

Quantitative real-time PCR was performed as previously described [26]. Briefly, all juvenile marine medaka fish were frozen and then placed into centrifuge tubes, with 5 fish in each tube. Total RNA was extracted using Trizol reagent, and RNA concentration was measured with a Q5000 UV-Vis spectrophotometer (Quawell, Quawell Technology, Inc., San Jose, CA, USA). cDNA was synthesized using the GoScript Reverse Transcription System kit (Promega, Madison, WI, USA) and followed by qPCR using GoTaq^®^ qPCR Master Mix kit (Promega, Madison, WI, USA). The 18s gene was used as a housekeeping gene to evaluate the relative transcript levels with $2^{-∆∆Ct}$ methods [27]. Specific primers for the target genes are listed in Table 1.

### 2.7. Data Analysis

Statistical analyses were performed using IBM SPSS Statistics 27.0 software for Windows (IBM SPSS Inc., Armonk, NY, USA), and graphical illustrations were designed using Origin 2024 software (Microcal Software Inc., Northampton, MA, USA). Normally distributed data were checked using Kolmogorov–Smirnov tests and Levene tests. A one-way analysis of variance followed by LSD tests was used to compare the differences between different treatment groups, and the differences were considered significant at *p* < 0.05.

## 3. Results

### 3.1. Effect of FEN and SMX on Development of Embryos of Marine Medaka

**Figure 1:**
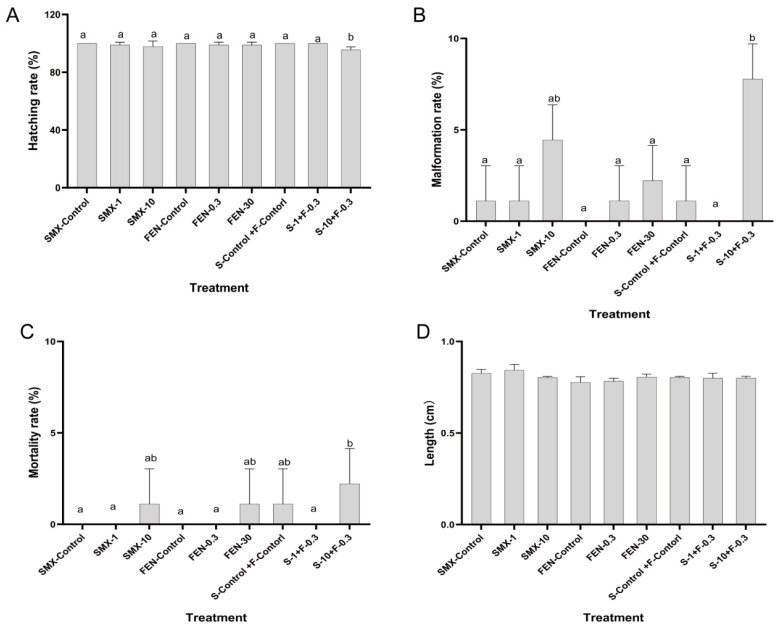
Effect of FEN and SMX on embryo development of marine medaka. (**A**) Hatching rate. (**B**) Malformation rate. (**C**) Mortality rate. (**D**) Length of juvenile marine medaka. Values are presented as mean ± SEM (*n* = 30). Different letters indicate statistical differences between groups (*p* < 0.05).

Figure 1A–C illustrate the embryonic development of marine medaka during exposure to FEN and SMX (0–12 dpf) (Table S1). Figure 1A demonstrates the effects of FEN and SMX on the hatching rate of marine medaka embryos. The S-10 + F-0.3 group exhibited a significantly lower hatching rate among all groups (*p* < 0.05). Figure 1B depicts the effects of FEN and SMX on the malformation rate in marine medaka embryos. The data indicate that the malformation rate in the SMX-10 group was significantly higher than that in the SMX control group (*p* < 0.05), and the S-10 + F-0.3 group exhibited the highest malformation rate among all groups (*p* < 0.05), suggesting that this combination promotes teratogenesis. Figure 1C shows the effects of FEN and SMX on the mortality rate of marine medaka embryos, with that of the S-10 + F-0.3 group being significantly higher than that of other groups (*p* < 0.05). Figure 1D represents the body length of juvenile marine medaka after a 15-day exposure period (15 dpf–30 dpf) (Table S1). The data reveal no significant differences between groups (*p* > 0.05).

### 3.2. Effects of FEN and SMX on Juvenile Marine Medaka H&E Sections

**Figure 2:**
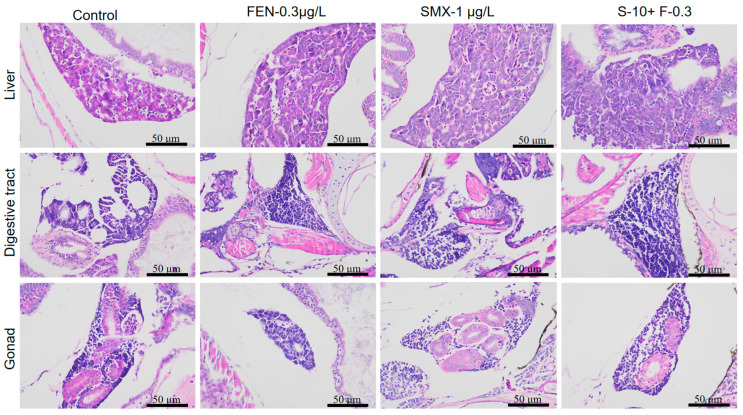
Histological observations of marine medaka after exposure to FEN and SMX.

From the tissue sections, there were no obvious pathological changes in the liver, heart, or digestive system among the groups (Figure 2). To further analyze the degree of damage, Image-Pro Plus software (Image-Pro Plus 6.0, Media Cybernetics, Inc., Rockville, MD, USA) was used for data processing to calculate cell quantity. There were no significant differences among each treatment (*p* > 0.05).

### 3.3. Transcriptomics

#### 3.3.1. Analysis of DEGs

**Figure 3:**
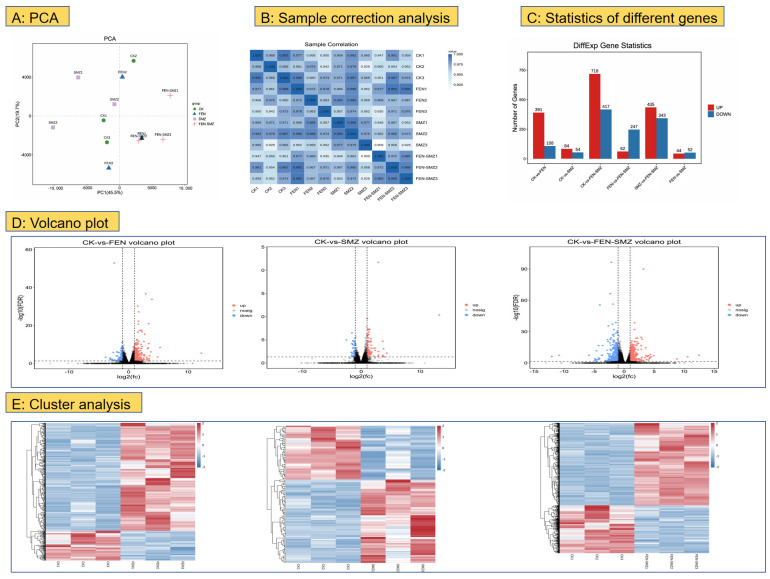
Analysis of DEGs regarding FEN and SMX exposure to juvenile marine medaka ((**A**), Principal component analysis (PCA). (**B**), Sample correction analysis. (**C**), Different expression genes. (**D**), Volcano plots of CK vs. FEN, CK vs. SMX, and CK vs. FEN−SMX. (**E**), Cluster analysis of CK vs. FEN, CK vs. SMX, and CK vs. FEN−SMX) (*n* = 3).

Transcriptome sequencing was conducted using Illumina paired-end sequencing technology, yielding 189,977,085 raw reads. After filtering low-quality data, the CK group, FEN group, SMX group, and F-S group obtained 46,309,507, 49,191,201, 51,747,931, and 42,676,188 clean reads, respectively. Using gene expression data from each sample, we randomly selected the expression levels of genes from pairs of samples, and the principal component analysis (PCA) results are presented (Figure 3A). Meanwhile, Pearson correlation coefficients for each sample pair were calculated, and inter-sample correlations were visualized as a heat map (Figure 3B).

Figure 3C shows the number of DEGs between CK vs. FEN, CK vs. SMX, and CK vs. F-S. As shown in the volcano plot (Figure 3D), the transcriptome sequencing of marine medaka samples after 15 days of exposure revealed that compared to the CK group, the FEN group had 499 differentially expressed genes (DEGs; 391 upregulated, 108 downregulated), the SMX group had 138 DEGs (84 upregulated, 54 downregulated), and the F-S group had 1135 DEGs (718 upregulated, 417 downregulated). Notably, the F-S group had significantly more DEGs than the combined total of the FEN and SMX groups (Figure S1). Additionally, DEGs were clustered, and a cluster analysis heat map was generated (Figure 3E).

#### 3.3.2. Gene Enrichment Analysis

**Figure 4:**
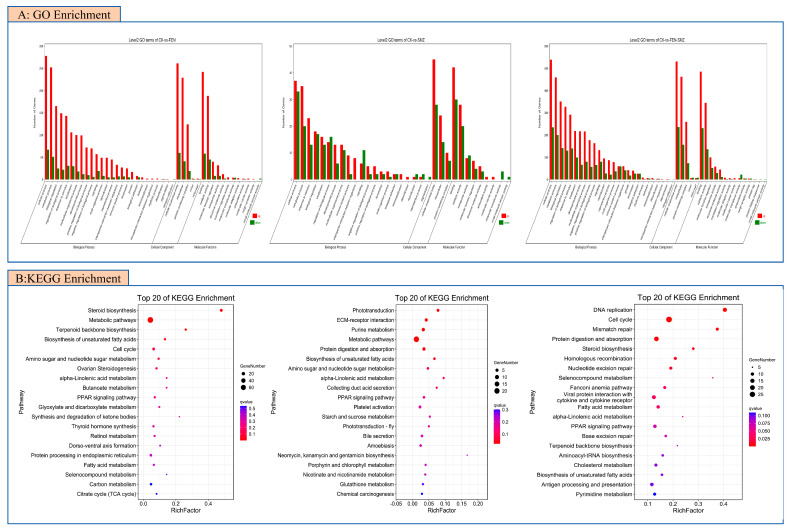
Gene enrichment analysis regarding FEN and SMX exposure to juvenile marine medaka ((**A**), Gene Ontology (GO) enrichment analysis of CK vs. FEN, CK vs. SMX, and CK vs. FEN−SMX; (**B**), Kyoto Encyclopedia of Genes and Genomes (KEGG) enrichment analysis of CK vs. FEN, CK vs. SMX, and CK vs. FEN−SMX) (*n* = 3).

The Gene Ontology (GO) enrichment analysis was performed with the GO database (http://geneontology.org, accessed on 16 October 2024). The results of GO enrichment analysis are shown in Figure 4A. In CK vs. FEN, the BPs (biological processes) significantly enriched by DEGs (differentially expressed genes) with *p* < 0.05 mainly include the cholesterol biosynthesis process, piRNA metabolic process, and steroid biosynthesis process. In CK vs. SMX, the significantly enriched BPs of DEGs mainly include the cysteine metabolic process, visual perception, and the (1-3)-β-D-glucan biosynthesis process. In CK vs. FS, the significantly enriched BPs of DEGs mainly include the mitotic cell cycle process, cell cycle process, and the G1/S transition of the mitotic cell cycle.

The Kyoto Encyclopedia of Genes and Genomes analysis was performed with the KEGG database (KEGG; https://www.kegg.jp/kegg/, accessed on 16 October 2024). To further determine the functions of differentially expressed genes (DEGs) and the specific pathways in which DEGs are enriched, we performed KEGG enrichment analysis on all DEGs from the treatment groups by comparing the control group with all treatment groups (Figure 4B). The KEGG enrichment analysis showed that compared with the control group, the numbers of pathways significantly enriched in growth and development-related pathways (*p* < 0.05) in the FEN group, SMX group, and FS group were 18, 12, and 21, respectively (Figure 3B: intergroup comparison bubble plot). In terms of the number of significantly enriched pathways related to the growth and development of juvenile marine medaka, we ranked the impact of pollutants on the growth and development of juvenile marine medaka as FS > FEN > SMX. To explore the differences in the effects of FEN and SMX alone and their combined action on the growth and development of juvenile marine medaka, KEGG enrichment analysis was performed on the DEGs between treatment groups. KEGG analysis identified important growth and development-related pathways (Q < 0.05). Compared with the FEN group, the FS group was significantly enriched in two growth and development-related pathways, namely the proteasome pathway and the NF-κβ signaling pathway (Figure S2). Compared with the FEN group, the SMX group was significantly enriched in one growth and development-related pathway, namely the steroid biosynthesis pathway. Meanwhile, we found that compared with the SMX group, the 18 pathways significantly enriched in the FS group were all related to various diseases or the immune system, including Staphylococcus aureus infection, the immune network for intestinal IgA production, and herpes simplex infection. Therefore, how SMX indirectly affects the growth and development of juvenile marine medaka through its impact on the immune system deserves further study.

### 3.4. Results of qRT-PCR

**Figure 5:**
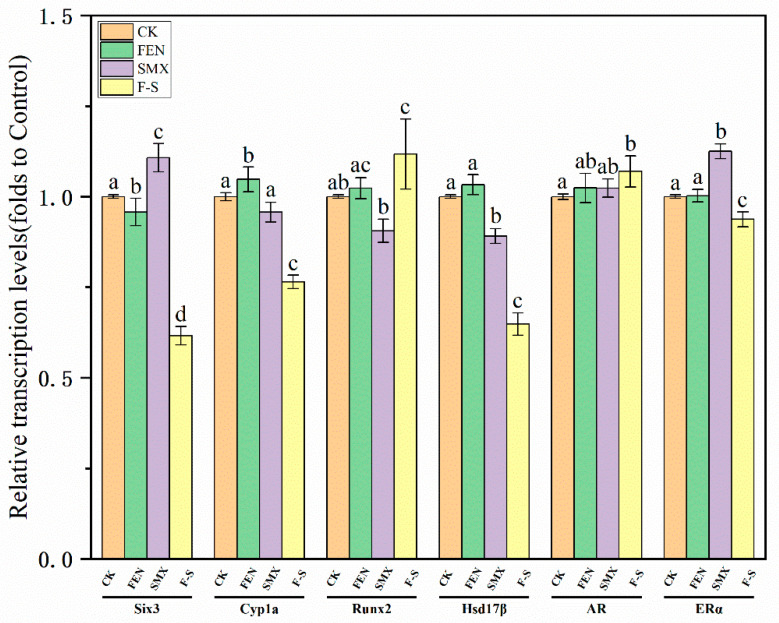
Effect of SMX and Fen on gene expression of marine medaka. Values are presented as mean ± SEM (*n* = 3). Different letters indicate statistical differences between each groups (*p* < 0.05).

Six candidate DEGs (Six3, Cyp1a, Runx2, Hsd17B, AR, ERα) were randomly selected to validate their expression levels using quantitative reverse transcription PCR (qRT-PCR) with 18srRNA as the inner reference gene. The expression of all six candidate genes showed high consistency and a strong correlation with their expression levels in the transcriptome results (Figure 5) [33,34]. The results of qRT-PCR analyses highlighted that the expression levels revealed by RNA-seq can accurately reflect the transcriptomic responses of marine medaka to FEN and SMX stresses.

## 4. Discussion

Increasing concerns regarding pesticide and antibiotic overuse have prompted extensive research, which has identified the toxic effects of new types of pesticide and antibiotic formulations on the early developmental stages of organisms (embryo and larva stages). Studies have found that a single exposure to FEN at a concentration of 3 μg·L^−1^ can inhibit the hatching of Japanese medaka and exert certain toxicity on embryonic development and survival [35]. Moreover, studies have also found that the toxicity of FEN to zebrafish follows the following order: embryos < larvae, juvenile fish < adult fish [29]. As the concentration increases, FEN will reduce the survival rate of zebrafish and increase the malformation rate [5,8]. This is inconsistent with our results, which may be due to differences in species or lower concentrations. Meanwhile, the exposure of zebrafish embryos to SMX at concentrations of 20 μg·L^−1^ significantly inhibits embryo hatching and significantly increases embryo mortality and malformation rates with 2 μg·L^−1^ [36]. In this study, the hatching rate of the S-10 + F-0.3 group was significantly lower than that of other groups, while the malformation and mortality rates of the combined group were significantly higher than those of other groups. We speculate that under single exposure, FEN and SMX at environmentally relevant concentrations have relatively low toxicity to marine medaka, but the combined toxicity shows a higher toxic effect, presenting a typical synergistic effect.

The exposure of zebrafish embryos to 12.5 mg·L^−1^ of FEN can induce brain damage in zebrafish [8]. In addition, studies have shown that exposing grass carp to 0.3 μg·L^−1^ SMX can cause adverse reactions such as the collapse of the columnar epithelial structure in the intestine, significant shortening of villus height and muscle layer thickness, and vacuolar degeneration of renal tubules and edema of renal tubular epithelial cells in the kidneys of grass carp [13,37]. However, in this study, no obvious pathological changes were found in the tissue sections. It is speculated that there are two possible reasons: first, the exposure concentration is low, and the marine medaka repaired itself through its own negative feedback effect; second, there may be differences between species and growth stages.

The F-S group exhibited significant transcriptome disruption (1135 DEGs), exceeding that of the groups exposed to FEN (499 DEGs) and SMX (138 DEGs), indicating a synergistic effect of their combined exposure at the molecular level. The KEGG enrichment analysis (*p* ≤ 0.05) of significantly altered pathways related to growth and development showed that 18, 12, and 21 pathways were disturbed in the FEN, SMX, and F-S groups, respectively.

In FEN group, the steroid biosynthesis pathway (P = 0.000000; Q = 0.000000) was significantly enriched with 12 DEGs, including the downregulation of *cyp24a1* and the upregulation of 11 DEGs (such as *Nsdhl*, *EBP*, and *cyp2r1*). Chen et al. (2024) have demonstrated that the knockout of the *EBP* gene in fish impairs cholesterol biosynthesis and estrogen levels, subsequently downregulating downstream genes (*sc5d*, *dhcr7*, and *dhcr24*) and body growth genes (*akt1* and *bmp2b*) [38]. These findings are consistent with the results, indicating that the upregulation of EBP detected in the FEN group may activate *akt1* and *bmp2b*, which may contribute to compensatory growth stimulation. In addition, the PPAR signaling pathway (P = 0.010561; Q = 0.257900) was enriched with five upregulated genes (such as *fads2*, *HMGCS1*, and *PLIN2*) and one downregulated gene (*ANGPTL4*). Due to the conserved role of *ANGPTL4* in mammals, its inhibition can enhance lipoprotein lipase (LPL) activity, promote triglyceride hydrolysis, and improve insulin sensitivity [39]. The downregulation of the *ANGPTL4* gene may enhance the efficiency of lipid and glucose metabolism, thereby promoting the growth of juvenile medaka under FEN stress.

In SMX group, the protein digestion and absorption pathway (P = 0.001560; Q = 0.031821) was significantly enriched with the upregulation of five genes, including *COL1A1* and *COL1A2*. Studies have found that the upregulation of *COL1A1* and *COL1A2* in fish is closely associated with enhanced collagen synthesis, TGF-β/Smad signaling, and muscle growth genes (such as *igf-1*, *myf5*, and *myhc*) [40]. Therefore, it is speculated that the upregulation of COL1A1 and COL1A2 can improve protein utilization and muscle development, thereby inducing a potential mechanism that promotes growth. Moreover, the knockout of the *hmox1a* and *hmox1b* genes during zebrafish development leads to developmental defects in zebrafish. The deletion of *hmox1a* inhibits the migration of macrophages to the wound, while the deletion of *hmox1b* has no such effect. It can be seen that *hmox* family genes play a key role in regulating the growth, development, and immune system of zebrafish [41]. These results are consistent with the enrichment of 21 upregulated DEGs including *hmox* and *phospho1* in the metabolic pathway (P = 0.000857; Q = 0.021857) found in this study. It is speculated that the upregulation of *hmox* genes may enhance antioxidant defense and immune function, thereby indirectly supporting growth. In addition, the *phospho1* gene plays a crucial regulatory role in the release of intracellular phosphate [42]. Phosphorus is an essential element for bone mineralization; the upregulation of this gene may increase the availability of phosphate for bone mineralization, thereby promoting the bone development and overall growth of fish in the SMX group.

In the F-S group, the fatty acid metabolism pathway (P = 0.001271; Q = 0.035929) was enriched with 11 DEGs, among which *CPT1A* was downregulated, while the other 10 DEGs (such as *fads2*, *Acat2*, and *Tecr*) were all upregulated. Studies have found that the upregulation of *fads2* promotes the biosynthesis of long-chain polyunsaturated fatty acids (LC-PUFA), which is crucial for the growth of juvenile fish [43]. The upregulation of fads2 was also observed in fish exposed to F-S, indicating that the development of fish in the FS group may be promoted by the increased production of LC-PUFA. *Hsd17β* is a key gene that regulates estrogen and androgen [44], and its downregulation indicates that the regulation of estrogen and androgen was disturbed in the F-S group. Six3 is one of the important genes for the development of the optic nerve in fish [45], and its downregulation suggests that the optic nerve development of juvenile marine medaka was under stress. In the cell cycle pathway (P = 0.000000; Q = 0.000000), 29 DEGs were significantly enriched, involving the downregulation of 3 DEGs (*CDKN2B*, *smad4*, *Gadd45g*) and the upregulation of 26 DEGs (*ORC1*, *mcm5*, *BUB1*, etc.). The transmission of the TGF-β signaling pathway is mediated by the homolog of *smad4* in fish [46], and this pathway usually inhibits cell cycle progression. For instance, by inhibiting the expression of c-Myc and Id family proteins, it reduces the possibility of cells entering a growth state [47]. Therefore, the downregulation of the *smad4* gene in juvenile medaka in the F-S group will reduce the activity of the TGF-β signaling pathway, thereby alleviating this inhibition and promoting cell proliferation and growth. Meanwhile, the DNA replication pathway (P = 0.000000; Q = 0.000000) was enriched with 15 upregulated DEGs such as *mcm5* and *pcna*. Studies have shown that the expression of *mcm5* in the zebrafish retina highly overlaps with the cell proliferation region and is consistent with the expression region of proliferating cell nuclear antigen (PCNA) [48], indicating that *mcm5* is a marker of proliferative capacity. Hence, we speculate that the upregulation of the *mcm5* gene in this experiment may synergize with PCNA to enhance cell proliferation, indirectly promoting the growth and development of juvenile medaka.

## 5. Conclusions

Due to the abuse of pesticides and antibiotics, it is crucial to investigate their potential combined toxicological effects in coastal waters. We exposed embryos and juveniles of marine medaka to environmentally relevant concentrations of FEN and SMX. This study found that single exposures to FEN or SMX did not significantly affect hatching, malformation, or mortality. Transcriptome analysis revealed enhancements in certain hormone synthesis, protein utilization, and muscle development, along with strengthened antioxidant defense and immune function, presenting a typical “hormesis effect”. However, in the F-S group, phenomena such as decreased hatching rate, increased teratogenesis, and elevated mortality were observed. Transcriptome analysis identified adverse effects including shortened cell cycles and the abnormal proliferation of retinal cells, indicating that the combined action of the two substances interferes with the development of juvenile marine medaka. Therefore, environmentally relevant concentrations of FEN and SMX may lead to a decline in the adaptability of fish during hatching and development. Although some “beneficial” phenomena may occur, the long-term “hormesis effect” will still endanger the survival of the species.

## Figures and Tables

**Table 1:** Primers for quantitative real-time PCR in juvenile marine medaka.

Gene	Sense Primer (5′-3′)	Antisense Primer (5′-3′)	GeneBank Number	Reference
*18s*	AACGCTGTGCTGCGTAGCCTCAATT	AGAAGAAGCCCCACTTTTCCTCGCA	DQ105650	[28]
*Six3*	GACTGACCCCGACTCAAGTG	CCGAGTCACTGTCCGTTACT	XM_024285009	[29]
*Cyp1a*	TCGTCGTTCTAAGTGGCAATGAAAC	AGAAAGAGCGCAATGCACTGTAGG		[30]
*Runx2*	TCTACGGACCTGAGCCCGTT	GGAGGAGGCGCCGTAGTAGA		[31]
*Hsd17B*	CGCTACCTCCACAAAGTTGTTGTC	AGTTCTGCCTCAACAGTTTCACCT	KF742611	[32]
*AR*	TTTGATGAACTGCGGACCTCCTAC	AACTGGTGCAATTTCCTCACAACC	KF742606	[32]
*ERα*	TCGCCGCTGTTGTGCTGTGATGTT	TCCTGGATCTGAGTGCGGGTCCGA	JF907629	[32]

## Data Availability

The data presented in this study are available on request from the corresponding author.

## Supplementary Materials

The following supporting information can be downloaded at: https://www.mdpi.com/article/10.3390/ani15182765/s1, Figure S1: Analysis of DEGs about FEN and SMX exposure to juvenile marine medaka (A, Volcano plots of FEN vs. FEN-SMX, SMX vs. FEN-SMX and FEN-SMX; B, Cluster analysis of FEN vs. FEN-SMX, SMX vs. FEN-SMX and FEN-SMX) (*n* = 3).; Figure S2:Gene enrichment analysis about FEN and SMX exposure to juvenile marine medaka (A, Gene Ontology (GO) enrichment analysis of FEN vs. FEN-SMX, SMX vs. FEN-SMX and FEN-SMX; B, Kyoto Encyclopedia of Genes and Genomes (KEGG) enrichment analysis of FEN vs. FEN-SMX, SMX vs. FEN-SMX and FEN-SMX) (*n* = 3). Table S1: Effect of FEN and SMX on embryos development of marine medaka.

## Abbreviations

The following abbreviations are used in this manuscript:

FEN	Fenvalerate
SMX	Sulfamethoxazole
AChE	Acetylcholinesterase
GO	Gene Ontology
KEGG	Kyoto Encyclopedia of Genes and Genomes
DEGs	Differentially expressed genes
qRT-PCR	Quantitative reverse transcription PCR
LC-PUFA	Long-chain polyunsaturated fatty acids
PCNA	Proliferating cell nuclear antigen
