# Supplementary material for: Transcriptomic Analysis Reveals Developmental Toxicity Induced by Environmentally Relevant Concentrations of Fenvalerate and Sulfamethoxazole in Embryo and Juvenile Marine Medaka (Oryzias melastigma, McClelland, 1839)
Source: Animals (Basel). 2025 Sep 22;15(18):2765. doi: 10.3390/ani15182765 (PMC12466638; doi:10.3390/ani15182765)
Supplement: Supplementary file 1 [file animals-15-02765-s001.zip › animals-3830537-supplementary.pdf]

Table S1. Effect of FEN and SMX on embryos development of marine medaka

| | | Hatching rate (%) | Malformation rate (%) | Mortality rate (%) | Length (cm） |
|---|---|---|---|---|---|
| SMX | Control | 100.00±0.00 | 1.11±1.92 | 0.00±0.00 | 0.83±0.02 |
| | 1 μg/L | 98.89±1.92 | 1.11±1.92 | 0.00±0.00 | 0.84±0.03 |
| | 10 μg/L | 97.78±3.85 | 4.44±1.92 | 1.11± 1.92 | 0.80±0.01 |
| FEN | Control | 100.00±0.00 | 0.00±0.00 | 0.00±0.00 | 0.78±0.03 |
| | 0.3 μg/L | 98.89±1.92 | 1.11±1.92 | 0.00±0.00 | 0.78±0.02 |
| | 30 μg/L | 98.89±1.92 | 2.22±1.92 | 1.11±1.92 | 0.81±0.02 |
| SMX+ FEN | Control | 100.00±0.00 | 1.11±1.92 | 1.11± 1.92 | 0.80±0.01 |
| | 1+0.3 μg/L | 100.00±0.00 | 0.00±0.00 | 0.00±0.00 | 0.80±0.03 |
| | 10+0.3 μg/L | 95.56±1.92 | 7.78±1.92 | 2.22±1.92 | 0.80±0.01 |

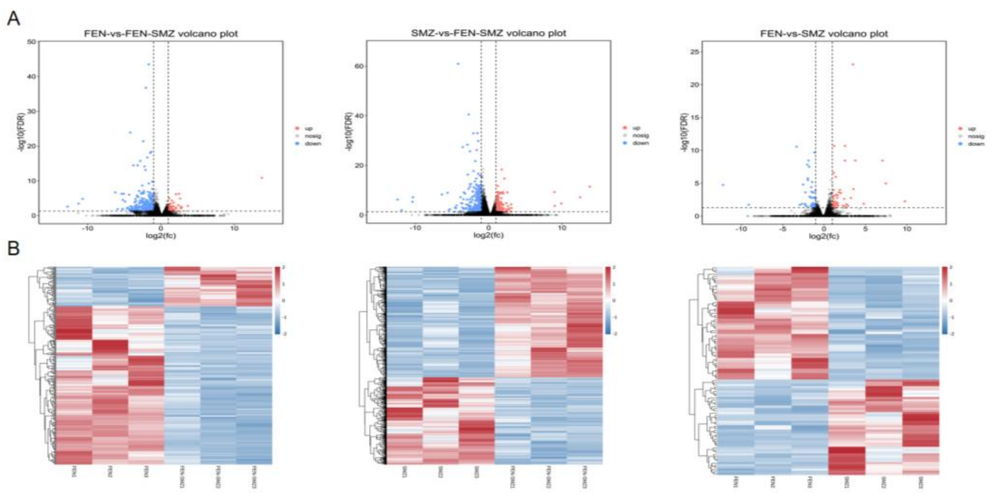


**Figure S1.** Analysis of DEGs about FEN and SMX exposure to juvenile marine medaka (A, Volcano plots of FEN vs FEN-SMX, SMX vs FEN-SMX and FEN-SMX; B, Cluster analysis of FEN vs FEN-SMX, SMX vs FEN-SMX and FEN-SMX) (*n*=3).

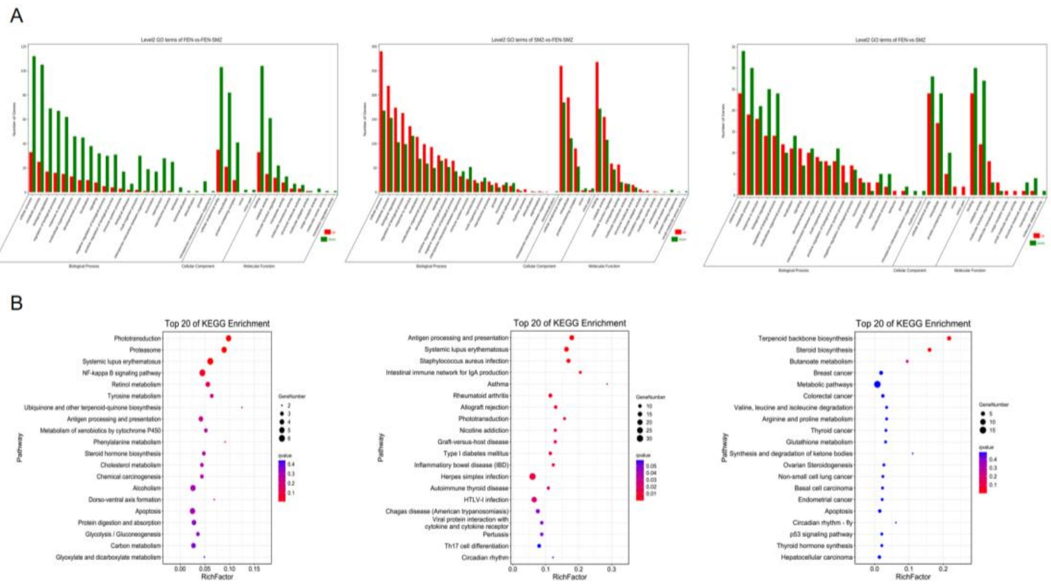


**Figure S2.** Gene enrichment analysis about FEN and SMX exposure to juvenile marine medaka (A, Gene Ontology (GO) enrichment analysis of FEN vs FEN-SMX, SMX vs FEN-SMX and FEN-SMX; B, Kyoto Encyclopedia of Genes and Genomes (KEGG) enrichment analysis of FEN vs FEN-SMX, SMX vs FEN-SMX and FEN-SMX )（*n*=3）.
